# Evaluation of an Automated System for the Counting of Microbial Colonies

**DOI:** 10.1128/spectrum.00673-23

**Published:** 2023-07-03

**Authors:** Elisa Heuser, Karsten Becker, Evgeny A. Idelevich

**Affiliations:** a Friedrich Loeffler-Institute of Medical Microbiology, University Medicine Greifswald, Greifswald, Germany; b Institute of Medical Microbiology, University Hospital Münster, Münster, Germany; JMI Laboratories

**Keywords:** colony counting, automatic counts, bacteria, yeast, agar

## Abstract

Counting of microbial colonies is a common technique employed in research and diagnostics. To simplify this tedious and time-consuming process, automated systems have been proposed. This study aimed to elucidate the reliability of automated colony counting. We evaluated a commercially available instrument (UVP ColonyDoc-It Imaging Station) in regard to its accuracy and potential time savings. Suspensions of Staphylococcus aureus, Escherichia coli, Pseudomonas aeruginosa, Klebsiella pneumoniae, Enterococcus faecium, and Candida albicans (*n* = 20 each) were adjusted to achieve growth of approximately 1,000, 100, 10, and 1 colony per plate, respectively, after overnight incubation on different solid media. Compared with manual counting, each plate was automatically counted by the UVP ColonyDoc-It with and without visual adjustment on a computer display. For all bacterial species and concentrations automatically counted without visual correction, an overall mean difference from manual counts of 59.7%, a proportion of isolates with overestimation/underestimation of colony numbers of 29%/45%, respectively, and only a moderate relationship (*R*^2^ = 0.77) with the manual counting were shown. Applying visual correction, the overall mean difference from manual counts was 1.8%, the proportion of isolates with overestimation/underestimation of colony numbers amounted to 2%/42%, respectively, and a strong relationship (*R*^2^ = 0.99) with the manual counting was observed. The mean time needed for manual counting compared with automated counting without and with visual correction was 70 s, 30 s, and 104 s, respectively, for bacterial colonies through all concentrations tested. Generally, similar performance regarding accuracy and counting time was observed with C. albicans. In conclusion, fully automatic counting showed low accuracy, especially for plates with very high or very low colony numbers. After visual correction of the automatically generated results, the concordance with manual counts was high; however, there was no advantage in reading time.

**IMPORTANCE** Colony counting is a widely utilized technique in the field of microbiology. The accuracy and convenience of automated colony counters are essential for research and diagnostics. However, there is only sparse evidence on performance and usefulness of such instruments. This study examined the current state of reliability and practicality of the automated colony counting with an advanced modern system. For this, we thoroughly evaluated a commercially available instrument in terms of its accuracy and counting time required. Our findings indicate that fully automatic counting resulted in low accuracy, particularly for plates with very high or very low colony numbers. Visual correction of the automated results on a computer screen improved concordance with manual counts, but there was no benefit in counting time.

## INTRODUCTION

Colony counting remains one of the most commonly used methods for determining the number of microorganisms in a sample (1, 2). Disregarding possible adhesion interactions and assuming sufficient dispersion, one vital cell of cultivable bacterial or fungal microorganisms being inoculated onto a solid nutrient medium gives rise to one colony after the incubation, typically overnight. Grown colonies are usually visible to the naked eye and can be counted. The term CFU, or typically CFU/milliliter, is therefore applied to indirectly describe the microbial concentration in the original suspension (3). The colony counting method is relatively simple, when small numbers of petri dishes with (semi-)solid agar media have to be counted, e.g., for confirmation of test inocula in antimicrobial susceptibility testing (1, 3). However, if performed manually, this process depends on the subjective skills of the person performing the task. In particular, the manual counting of colonies can become a laborious and tiring task when numerous plates are processed. As an example, determination of minimal bactericidal concentrations or, particularly, evaluation of rapidity of bactericidal effect in time-kill experiments necessitates that multiple samples of serial dilutions be investigated for different drug concentrations, time points, replicates, and other issues (4, 5). Moreover, high colony numbers may lead to false results because often only parts of a given plate are being counted. Automated colony counters have been developed for handling high plate volumes (6). However, the accuracy of counting should remain the highest priority (6, 7).

In this study, we evaluated a commercially available instrument for automated counting of microbial colonies in regard to its accuracy and potential time saving. For this, we challenged the device with various bacterial and fungal species possessing different morphological characteristics. Moreover, different agar types were included.

(Parts of this study were presented at the Annual Meeting of the German Society for Hygiene and Microbiology [DGHM] in 2022, Berlin, Germany [136/DKMV]).

## RESULTS

### Evaluation of accuracy.

In comparison with the manual counts, the overall mean difference of the automated count without and with visual correction was 59.7% and 1.8%, respectively, for all bacterial species and concentrations tested on tryptic soy agar (TSA) (Table 1). For Candida albicans, the mean difference for the automated count was 71.4% without visual correction and 2.8% with visual correction (Table 2). For two bacterial species tested on Columbia blood agar (CBA), the mean differences of the automated count without visual correction and the automated count with visual correction were 18.3% and 1.0% (Table 3), respectively. The corresponding results for each microbial concentration tested are demonstrated in Tables 1 to 3.

**TABLE 1:** Mean difference of automated counts with and without visual correction for bacterial species grown on tryptic soy agar, compared with manual count

Species (no. of strains)	Expected approx concn in suspension, CFU/mL	Expected approx colony no./plate, *n*	Real colony no./plate,^*a*^ *n*			Mean difference from manual count (%)	
			Mean	Median	Range	Automated count without visual correction	Automated count with visual correction
Overall total (all species and concns)			184	16	0–1,248	59.7	1.8

Staphylococcus aureus (*n* = 20)	All concns	All colony no.	211	16	0–1,248	58.7	0.9
	10^5^	1,000	753	805	97–1,248	39.2	2.6
	10^4^	100	84	94	5–137	9.7	0.7
	10^3^	10	8	8	0–17	27.7	0.4
	10^2^	1	1	0	0–2	158.3	0.0

Enterococcus faecium (*n* = 20)	All concns	All colony no.	182	27	0–1,068	33.1	2.9
	10^5^	1,000	642	609	442–1,068	27.6	1.8
	10^4^	100	76	72	36–137	9.8	2.1
	10^3^	10	8	7	4–18	19.3	2.8
	10^2^	1	1	1	0–4	75.8	5.0

Escherichia coli (*n* = 20)	All concns	All colony no.	184	27	0–884	70.9	1.6
	10^5^	1,000	675	630	329–884	78.2	3.9
	10^4^	100	94	94	37–127	29.4	2.0
	10^3^	10	10	10	3–16	20.3	0.4
	10^2^	1	1	1	0–3	155.6	0.0

Klebsiella pneumoniae (*n* = 20)	All concns	All colony no.	153	13	0–883	73.2	1.1
	10^5^	1,000	513	575	99–883	66.0	2.2
	10^4^	100	71	79	3–129	18.8	2.0
	10^3^	10	9	9	0–16	27.4	0.0
	10^2^	1	1	1	0–5	180.6	0.0

Pseudomonas aeruginosa (*n* = 20)	All concns	All colony no.	192	29	0–993	62.7	2.5
	10^5^	1,000	654	633	215–993	51.0	3.3
	10^4^	100	85	86	42–157	16.2	5.0
	10^3^	10	8	7	1–15	42.1	1.8
	10^2^	1	1	0	0–4	141.7	0.0

a According to the manual counting.

**TABLE 2:** Mean difference of automated counts with and without visual correction for Candida albicans (*n* = 20) grown on Sabouraud agar, compared with manual count

Expected approx concn in suspension, CFU/mL	Expected approx colony no./plate, *n*	Real colony no./plate,^*a*^ *n*			Mean difference from manual count (%)	
		Mean	Median	Range	Automated count without visual correction	Automated count with visual correction
All concns	All colony no.	270	30	0–1,578	71.4	2.8
10^5^	1,000	1,076	1,142	503–1,578	41.4	5.2
10^4^	100	181	194	37–277	20.4	0.9
10^3^	10	22	24	5–40	16.0	2.1
10^2^	1	3	3	0–7	207.4	3.0

a According to the manual counting.

**TABLE 3:** Mean difference of automated counts with and without visual correction for Staphylococcus aureus and Enterococcus faecium grown on Columbia blood agar, compared with manual count

Species (no. of strains)	Expected approx concn in suspension, CFU/mL	Expected approx colony no./plate, *n*	Real colony no./plate,^*a*^ *n*			Mean difference from manual count (%)	
			Mean	Median	Range	Automated count without visual correction	Automated count with visual correction
Overall total (both species and all concns)			175	20	0–1,061	18.3	1.0

Staphylococcus aureus (*n* = 20)	All concns	All colony no.	182	20	0–1,061	23.3	0.7
	10^5^	1,000	629	610	304–1,061	40.2	0.9
	10^4^	100	91	87	19–134	14.8	1.7
	10^3^	10	9	9	0–21	13.0	0.0
	10^2^	1	1	1	0–3	25.0	0.0

Enterococcus faecium (*n* = 20)	All concns	All colony no.	168	24	0–839	13.2	1.2
	10^5^	1,000	582	559	337–839	16.0	2.5
	10^4^	100	79	75	35–121	6.3	0.9
	10^3^	10	8	8	1–13	5.3	1.6
	10^2^	1	1	1	0–2	25.0	0.0

a According to the manual counting.

**FIG 1:**
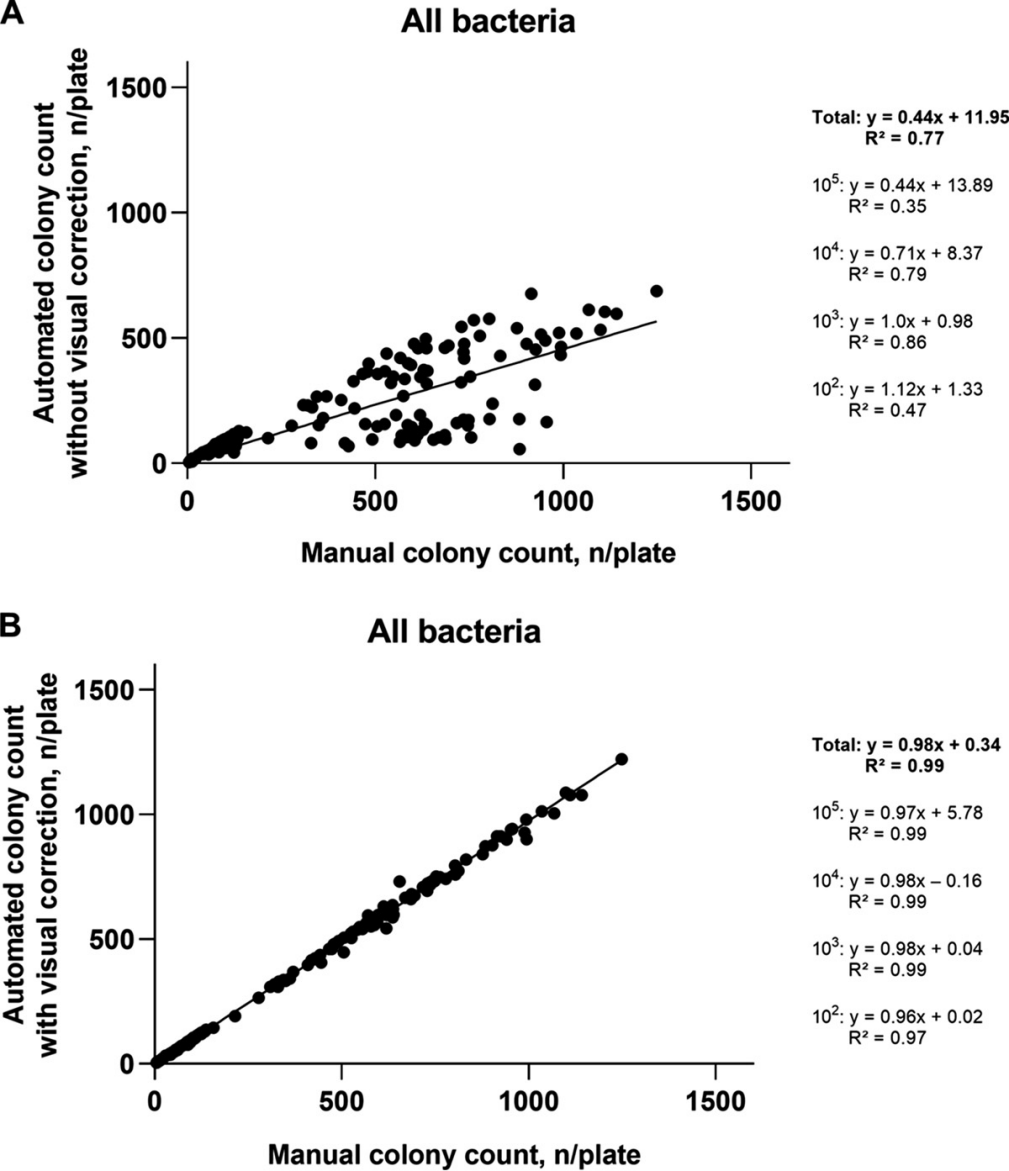
Relationship between automated colony counts and manual counting for all tested bacterial species on tryptic soy agar. (A) Automated colony counts without visual correction. (B) Automated colony counts with visual correction. 10^5^, 10^4^, 10^3^, and 10^2^, expected approximate concentration levels in suspensions (CFU/milliliter) which after plating onto agar result in the expected approximate colony numbers of 1,000, 100, 10, and 1 per plate, respectively.

**FIG 2:**
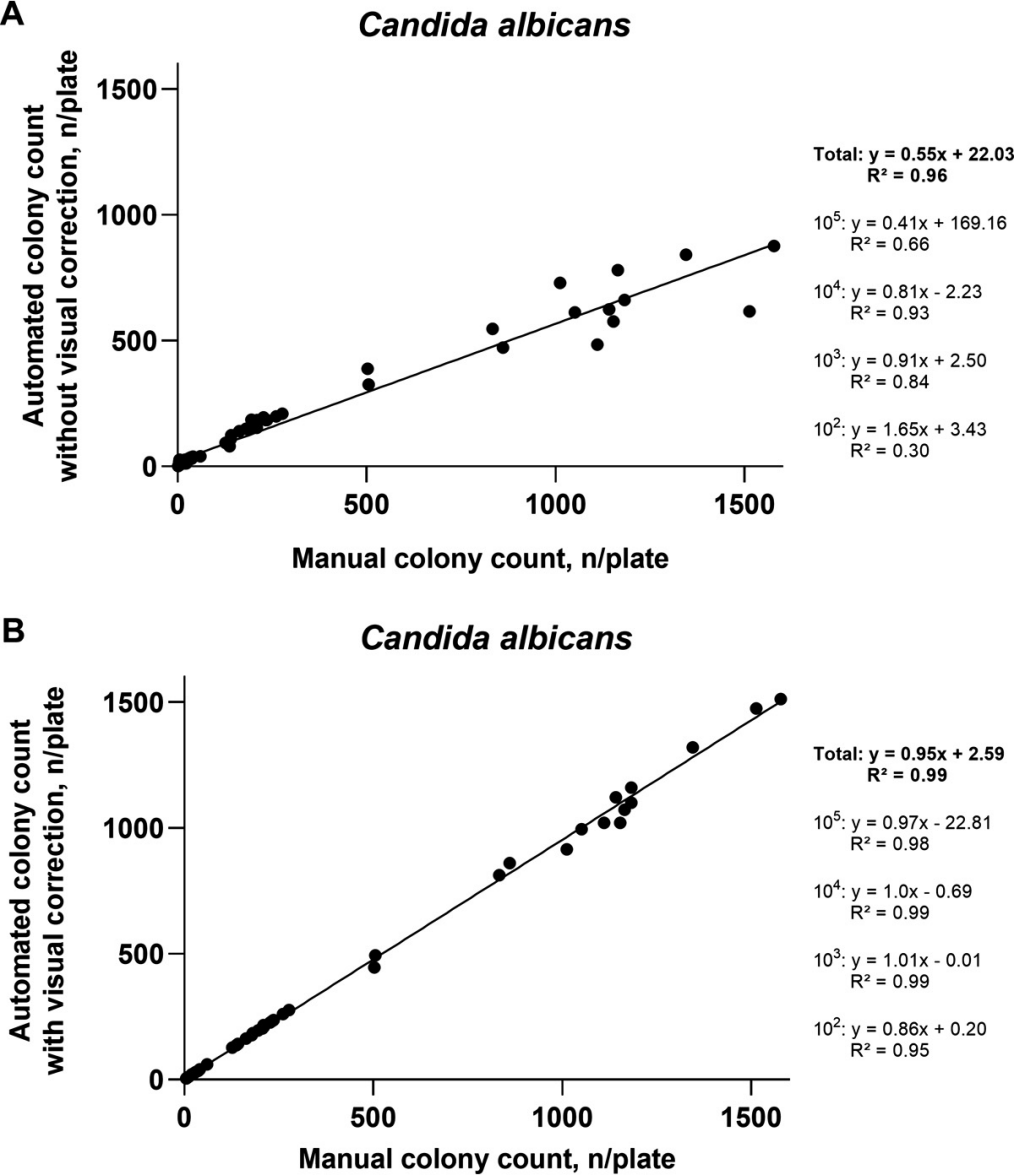
Relationship between automated colony counts and manual counting for Candida albicans on Sabouraud agar. (A) Automated colony counts without visual correction. (B) Automated colony counts with visual correction. 10^5^, 10^4^, 10^3^, and 10^2^, expected approximate concentration levels in suspensions (CFU/milliliter) which after plating onto agar result in the expected approximate colony numbers of 1,000, 100, 10, and 1 per plate, respectively.

**FIG 3:**
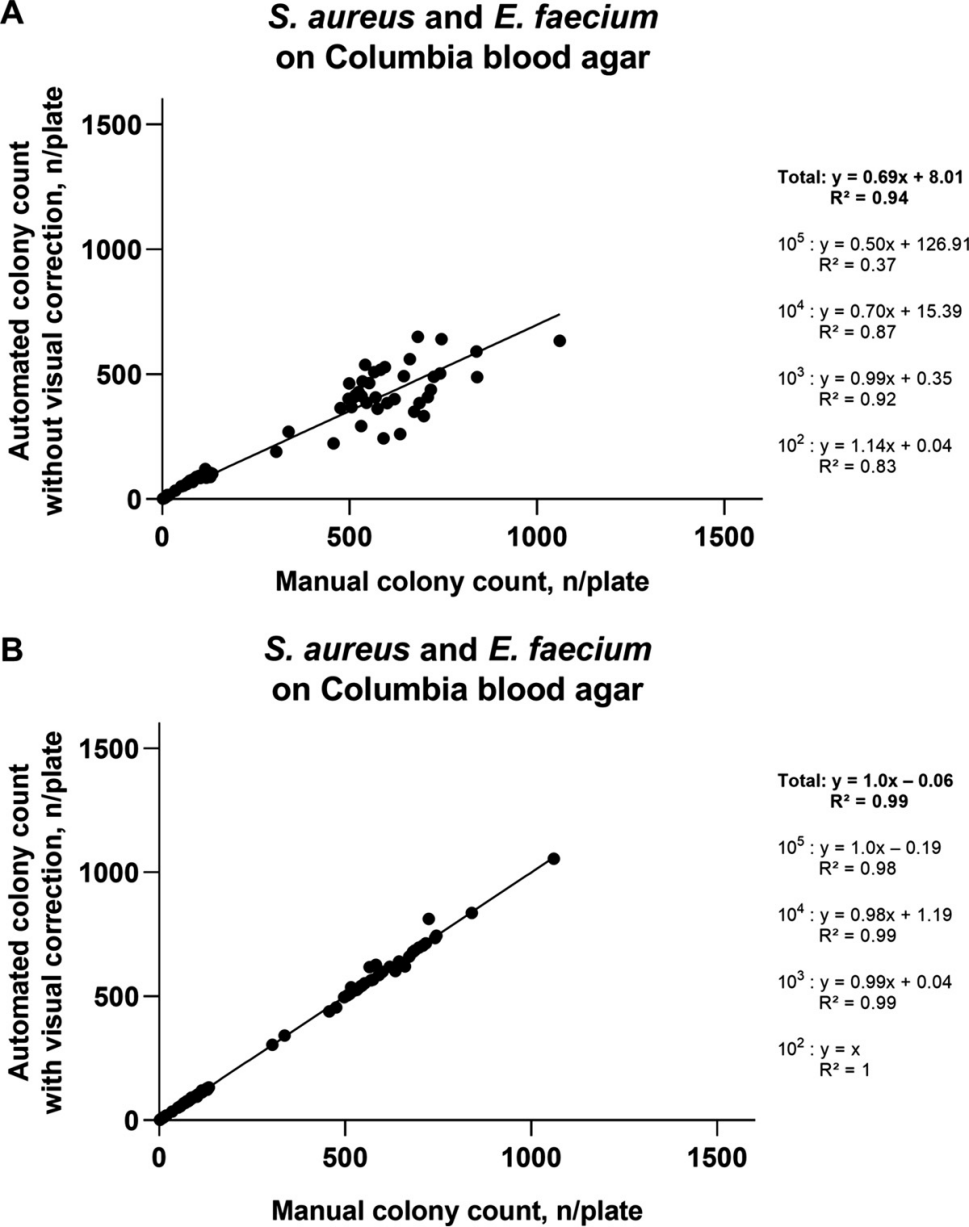
Relationship between automated colony counts and manual counting for S. aureus and E. faecium on Columbia blood agar. (A) Automated colony counts without visual correction. (B) Automated colony counts with visual correction. 10^5^, 10^4^, 10^3^, and 10^2^, expected approximate concentration levels in suspensions (CFU/milliliter) which after plating onto agar result in the expected approximate colony numbers of 1,000, 100, 10, and 1 per plate, respectively.

For all bacterial species and all concentrations counted on TSA, the automated counts without correction showed only a moderate relationship (*R*^2^ = 0.77) with manually acquired results (Fig. 1A), while machine counts with correction were strongly related (*R*^2^ = 0.99) with manual results (Fig. 1B). A similar trend was observed for C. albicans (*R*^2^ = 0.96 for automated counts without correction and *R*^2^ = 0.99 for the counts with correction) (Fig. 2A and B) and for two bacterial species investigated on CBA (*R*^2^ = 0.94 for automated counts without correction and *R*^2^ = 0.99 for the counts with correction) (Fig. 3A and B). The regression equations and *R*^2^ values for individual microbial concentrations are given in Fig. 1 to 3.

**FIG 4:**
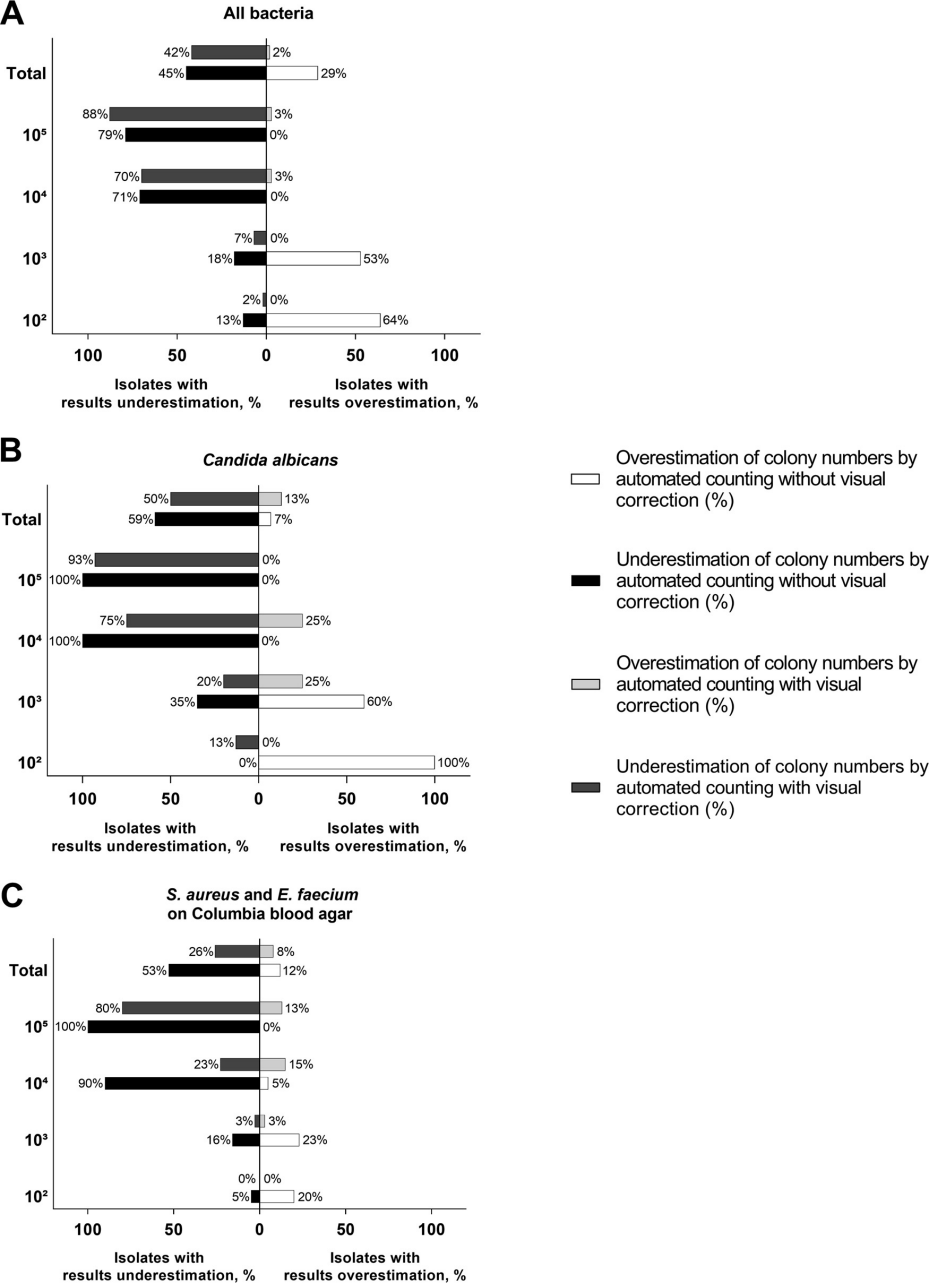
Percentage of isolates with overestimation and underestimation of colony numbers by automated counting without visual correction and with visual correction, compared with manual counting. (A) For all tested bacterial species on tryptic soy agar. (B) For Candida albicans on Sabouraud agar. (C) For S. aureus *and* E. faecium on Columbia blood agar.

For automated counts performed for all bacterial species and concentrations on TSA without visual correction and with visual correction, the proportions of isolates with overestimation/underestimation of colony numbers were 29%/45% and 2%/42%, respectively. The number of colonies on a plate was overestimated/underestimated in 7%/59% and 13%/50% of C. albicans isolates without visual adjustment and with visual adjustment of automated counting, respectively. For the counting of bacteria on CBA, overestimation/underestimation of colony numbers was observed in 12%/53% and 8%/26% of isolates, when automatically generated results were not visually adjusted and when they were visually adjusted, respectively. The proportions of isolates with overestimation or underestimation of counts for each microbial concentration are shown in Fig. 4A to C.

### Assessment of counting time.

**FIG 5:**
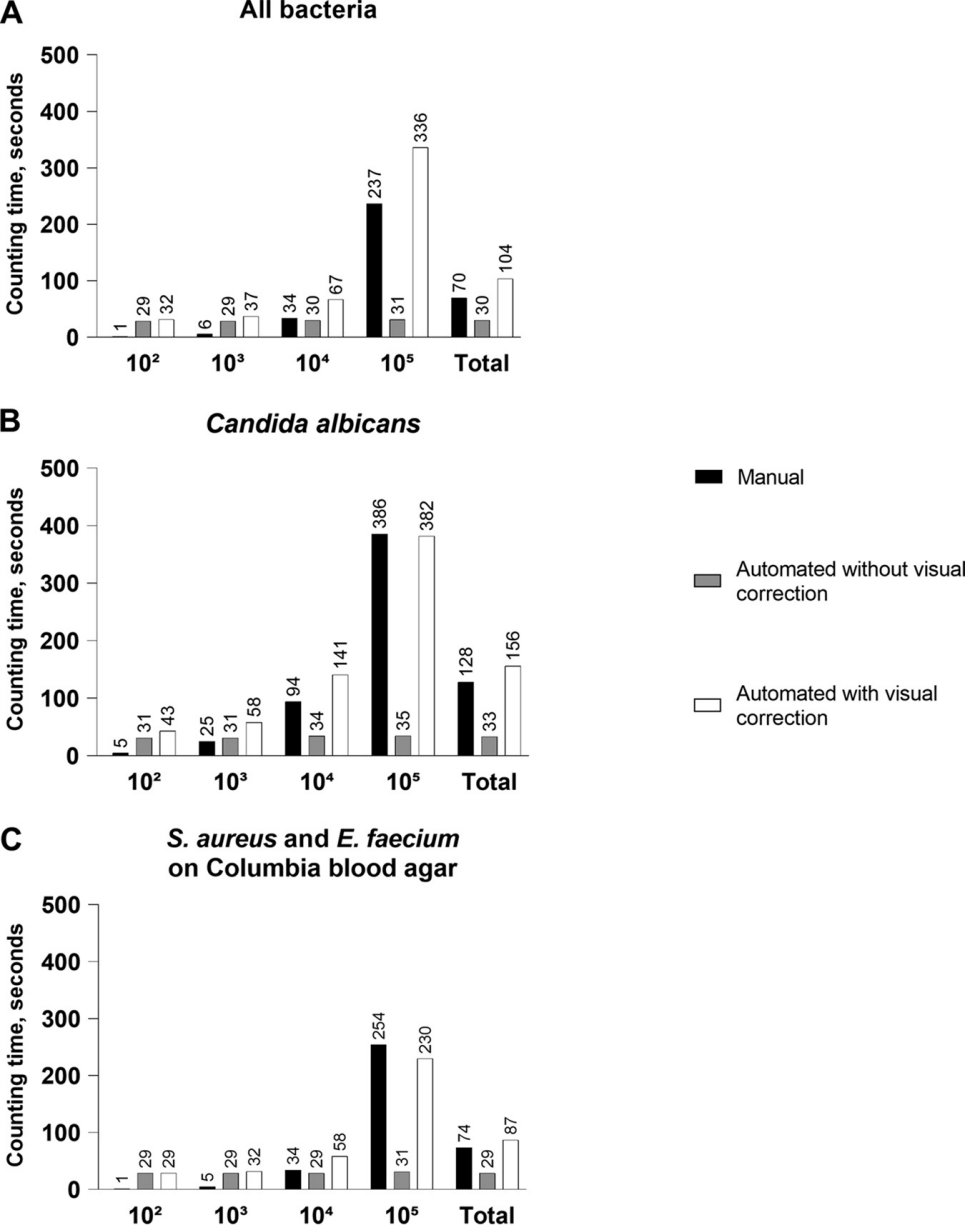
Time for counting of colony numbers by automated counting without visual correction and with visual correction, compared with manual counting. (A) For all tested bacterial species on tryptic soy agar. (B) For Candida albicans on Sabouraud agar. (C) For S. aureus and E. faecium on Columbia blood agar.

The mean time needed for manual counting, automated counting without visual correction, and automated counting with visual correction of bacterial colonies on TSA was 70 s, 30 s, and 104 s, respectively, for all concentrations tested (Fig. 5A). For counts of C. albicans colonies, corresponding times of 128 s, 33 s, and 156 s were recorded (Fig. 5B). These counting times (manual, automated without visual correction, and automated with visual correction) amounted to 74 s, 29 s, and 87 s for the two bacterial species grown on CBA, respectively (Fig. 5C). The time spent for counting of each microbial concentration is shown in detail in Fig. 5A to C.

## DISCUSSION

The counting of microbial colonies on agar plates is a commonly used method in laboratories but is very time-consuming depending on the number of plates. This procedure could be simplified and shortened if the counting was automated. Solutions range from free or low-cost software that can be used with a smartphone camera (6–9) to advanced commercially available instruments (6, 10–12).

The study’s main intention was to elucidate the question of whether a modern automatic colony counter is reliable enough to eventually replace tedious manual counting. With this aim, we performed a thorough evaluation of the UVP ColonyDoc-It Imaging Station (Analytik Jena US). We were unable to find any publication evaluating this instrument for counting microbial colonies. However, there are reports of using it for counting colonies of mammalian cell cultures (13, 14).

In total, 640 agar plates were evaluated in our study. We included microbial species that are common in clinical diagnostics and are frequently used in research. These species represented Gram-positive and Gram-negative bacteria as well as yeasts to reflect the broad range in colony shape, size, and color. Twenty isolates of each species were included as the colony morphology may considerably vary within the same species. All isolates were tested on transparent agar, which was TSA for bacteria and Sabouraud agar for yeasts. Additionally, Staphylococcus aureus and Enterococcus faecium were tested on CBA to investigate whether the specific hemolysis zones formed with these species around the colony on a blood-containing medium or/and the blood-containing medium itself may limit the performance of the instrument.

The experiments revealed low accuracy of the fully automatic counts, especially for the plates with very high or very low bacterial density (Tables 1 to 3; Fig. 1 to 4). After visual editing of the automated counts through the software interface using saved and labeled pictures, correlation with manual counts was considerably improved (Tables 1 to 3; Fig. 1 to 4). As in other reports (6), the latter improvement in accuracy was at the expense of considerable time investment for human intervention (Fig. 5).

The accuracy of and the time spent for the counting of C. albicans (Table 2, Fig. 2, Fig. 4B, and Fig. 5B) were similar to those documented for bacterial species grown on TSA (Table 1, Fig. 1, Fig. 4A, and Fig. 5A). Thus, counting of yeasts does not seem to represent a specific challenge for automatic systems. Also, there were no remarkable differences in performance when S. aureus and E. faecium, which on blood-containing media produce complete hemolysis or alpha-hemolysis, respectively, were tested on CBA (Table 3, Fig. 3, Fig. 4C, and Fig. 5C), compared with the testing on TSA. A previous study demonstrated accurate discrimination of alpha-hemolytic colonies of Streptococcus pneumoniae on CBA with a novel segmentation algorithm (15). Another recent study reported poor recognition of hemolytic colonies by a professional automated counter and by phone applications (6).

Underestimation of colony numbers by the automated system was frequently observed at high colony densities, whereas overestimation of counts was often observed at low colony densities. A similar trend was reported from early (16, 17) and more recent (7, 10, 12, 18, 19) studies performed with other systems.

The counting errors occurring with plates densely covered with colonies were most commonly due to confluent colonies which could not be discriminated by the machine. However, we also observed that, in the setting of very high colony numbers, individually located discrete colonies were occasionally not recognized. Problematic separation of the overlapping colonies by the automatic algorithms has been reported by many authors as one of the most important sources of errors (11, 17, 19). On the other hand, the instrument detected not only the real colonies but also scratches and air bubbles in the agar, imprints on the petri dish, and light reflections. These errors, which were also found in other studies (6, 7, 9, 10, 16, 17, 20), led to the false-positive results, particularly at low concentrations.

Early descriptions of automated colony counters appeared decades ago (16, 17). At that time, concern had been expressed regarding discrepancies with manual counting (17), which remains the gold standard method for colony enumeration nowadays (7, 8, 18). In our study and in other recent publications with contemporary systems (6, 7), such concerns regarding the accuracy of fully automatic counting continue to exist. In 2022, Moucka et al. evaluated four smartphone colony counter applications and found that the performance varied from poor to good, depending on the application used (7). The authors concluded that none of the evaluated products can fully replace manual counting but applications can be used to provide an estimation of the CFU numbers or semiquantitative results (7). Young et al. demonstrated compromised accuracy of counting systems unless considerable time was invested for human adjustment (6).

In general, our study revealed insufficient accuracy of fully automated counting. It may provide only a very crude estimation of colony numbers which would satisfy only a limited number of applications. When automated counts were visually adjusted, correlation with manual counts was considerably improved. Still, discrepancies with manual results were frequently observed. Automated counting with visual correction of results may be a useful approach, if the task requirements can be fulfilled with the approximation of colony numbers. Due to the time expenditure for human adjustment, however, there was no advantage in reading time compared with manual counting. Possible negative effects of longer working hours at digital displays must also be taken into account (21).

Manual colony enumeration remains indispensable when the goal is to achieve the highest accuracy of counting. For many applications, the counting needs to be highly exact in the higher and the lower range of colony numbers. To illustrate the latter requirement, the investigation of bactericidal activity can be taken as an example. The guideline for determination of bactericidal activity of antimicrobial drugs specifies the final inoculum used in the test to be 5 × 10^5^ CFU/mL, and the bactericidal activity is defined as killing 99.9% of the initial inoculum (4). Thus, a cutoff of 5 × 10^2^ CFU/mL divides evidence from no evidence of bactericidal activity; that is exactly 5 colonies on a petri dish if 10-μL samples are spread. Thus, the highest accuracy of colony counting is of paramount importance for this application.

Several authors tried to define acceptance criteria for the accuracy of automated counting systems (6, 11, 19, 22–24). The accuracy was defined as acceptable if the automated counts were within an 0.5-log_10_ range of the manual count (11, 23, 24), if 90% of the automated counts were within 10% of the corresponding manual count (22), or if the counting error was not higher than 15 to 20% (6, 19). In our opinion, no definition of accuracy will match the requirements of every application. Rather, it will remain the investigator’s responsibility to determine whether the system’s accuracy is appropriate for a particular application.

The limitation of our study is that manual counting, with which other results were compared as with reference values, was performed by one investigator. However, a recent study indicated that variance was low when manual CFU counting was done by three independent individuals (7). An important source of errors with manual enumeration is counting only a portion of the plate area and then multiplying by the total number of sectors (6, 15, 16). Obviously, this practice should not be used when exact results are needed or when the manual results are used as a reference for evaluation of automated systems. Otherwise, manual reading is straightforward, and even such phenomena as partly confluent colonies or colonies in the rim area can easily be recognized. In our study, the same microbiology scientist completely counted all plates throughout the study to ensure uniform reading. The same holds true for feeding the plates into the instrument prior to the automated counting and for visual adjustment of colony numbers on the screen after automated counting. While the fully automated counting is not affected by an operator, the accuracy of the visual adjustment may be lower if performed by a less experienced user. Another study limitation is that it was naturally not feasible to test all clinically relevant species or microorganism groups, e.g., anaerobes.

In conclusion, the fully automatic counting showed low accuracy, especially for plates with very high or very low colony numbers. After visual correction of the automatically generated results, the correlation with manual counts was high; however, there was no advantage in reading time. There were no appreciable differences in accuracy between the different agar types and microorganisms. It seems that even in the current era of technological advances in the life sciences, reliable colony counting by automated systems remains challenging and does not reach the reliability of the human eye (and judgment). Automatic counting provides an approximation of colony numbers but cannot replace manual counting if high accuracy is required. Significant improvement in accuracy of automatic colony counting would considerably expand the range of potential implementations, including academic studies, industrial research, and clinical diagnostics.

## MATERIALS AND METHODS

### Microbial strains.

For the counting experiments, which took place between April and September 2022, five different bacterial species and one yeast species were used. Twenty consecutively collected clinical strains of each of the following species were included: Staphylococcus aureus, Escherichia coli, Pseudomonas aeruginosa, Klebsiella pneumoniae, Enterococcus faecium, and Candida albicans. Only one isolate per patient was eligible.

### Preparation of inoculum.

The strains were cultivated overnight on Columbia blood agar (BBL Columbia agar with 5% sheep blood; BD, Franklin Lakes, NJ, USA) at 35 ± 1°C in ambient air. A colony from the overnight culture was adjusted to an 0.5 McFarland turbidity standard in 2 mL 0.9% NaCl to obtain a microbial concentration of approximately 1 × 10^8^ CFU/mL for the bacteria and approximately 1 × 10^6^ CFU/mL for C. albicans. Afterward, the cultures were serially diluted 1:10 in 0.9% NaCl to produce suspensions containing approximately 10^5^, 10^4^, 10^3^, and 10^2^ CFU/mL. From each dilution of bacterial suspensions, 10-μL samples were plated onto a 90-mm tryptic soy agar (TSA) plate (BD Diagnostics, Heidelberg, Germany), anticipating the growth of approximately 1,000, 100, 10, and 1 colony per plate, respectively. The suspensions were evenly spread using a bent inoculation loop as a cell spreader. For E. faecium and S. aureus, these serial dilutions were additionally plated on a 90-mm Columbia blood agar plate. For the yeasts, 10-μL samples were plated onto 90-mm Sabouraud agar plates (Sabouraud agar with gentamicin and chloramphenicol; BD, Heidelberg, Germany). Incubation was performed for 18 to 20 h at 35 ± 1°C.

### Automated versus manual colony counting.

For each plate, the counting was performed with three different procedures: (i) fully automated counting by the instrument without any visual adjustment, (ii) automated counting by the instrument with additional visual adjustment on a computer display, and (iii) manual counting. Each strain was tested once with each of these procedures.

Automated counting was performed by the UVP ColonyDoc-It Imaging Station (Analytik Jena US, Upland, CA, USA) using the VisionWorks Capture and Analysis software 9.1 (Analytik Jena US). All plate pictures were captured with the high-quality-standard setting, with the doors of the imaging station closed, with overhead white light, and with a black background plate. The agar plates were automatically counted with the agar surface facing up toward the camera with plate lids removed. These fully automatically obtained results were recorded.

Subsequently, the pictures of plates with the labeled results of the fully automated colony counting were visually observed by the investigator on a computer display. In the case of obvious deviations, the results were manually adjusted through the software interface and documented.

For the manual counting, the plates were placed with the lid closed onto the illuminated stage of a manual colony counter (SC6+; Cole-Parmer, St Neots, United Kingdom) and counted visually from the back without any magnifying aids. Due to the limited transparency of the Columbia blood agar, these plates were additionally checked from the front side without a lid.

The time needed for each counting procedure was recorded.

### Statistical analysis.

Regression analysis was performed with each microbial concentration for the automated colony counts without visual correction and for the automated counts with visual correction. The results of manual counting were used as reference values. Statistical and graphical analysis was performed with GraphPad Prism 9 (GraphPad Software, San Diego, CA, USA).

## References

[1] Clinical and Laboratory Standards Institute. 2018. Methods for dilution antimicrobial susceptibility tests for bacteria that grow aerobically; approved standard, 11th ed, M07-A11. Clinical and Laboratory Standards Institute, Wayne, PA.

[2] Herten M, Bisdas T, Knaack D, Becker K, Osada N, Torsello GB, Idelevich EA. 2017. Rapid in vitro quantification of S. aureus biofilms on vascular graft surfaces. Front Microbiol 8:2333. doi:10.3389/fmicb.2017.02333. 29259580 PMC5723318

[3] International Organization for Standardization. 2019. 20776-1. Susceptibility testing of infectious agents and evaluation of performance of antimicrobial susceptibility test devices - part 1: broth micro-dilution reference method for testing the in vitro activity of antimicrobial agents against rapidly growing aerobic bacteria involved in infectious diseases, 2nd ed. International Organization for Standardization, Geneva, Switzerland.

[4] National Committee for Clinical Laboratory Standards. 1999. Methods for determining bactericidal activity of antimicrobial agents; approved guideline, M-26A. National Committee for Clinical Laboratory Standards, Wayne, PA.

[5] Heuser E, Becker K, Idelevich EA. 2022. Bactericidal activity of sodium bituminosulfonate against Staphylococcus aureus. Antibiotics (Basel) 11:896. doi:10.3390/antibiotics11070896. 35884150 PMC9311858

[6] Young LM, Rieman DJ, Walden L, Motz VA. 2018. In search of a counter you can count on: relative efficacy of human visual and automated colony counting. Lett Appl Microbiol 66:188–193. doi:10.1111/lam.12851. 29341168

[7] Moucka M, Muigg V, Schlotterbeck AK, Stoger L, Gensch A, Heller S, Egli A. 2022. Performance of four bacterial cell counting apps for smartphones. J Microbiol Methods 199:106508. doi:10.1016/j.mimet.2022.106508. 35691441

[8] Austerjost J, Marquard D, Raddatz L, Geier D, Becker T, Scheper T, Lindner P, Beutel S. 2017. A smart device application for the automated determination of E. coli colonies on agar plates. Eng Life Sci 17:959–966. doi:10.1002/elsc.201700056. 32624845 PMC6999497

[9] Geissmann Q. 2013. OpenCFU, a new free and open-source software to count cell colonies and other circular objects. PLoS One 8:e54072. doi:10.1371/journal.pone.0054072. 23457446 PMC3574151

[10] Mahapatra AK, Nguyen CN, Kannan G, Harris DL. 2009. Evaluation of an IUL Flash & Go automated colony counter. Agric Eng Int CIGR J XI:1368.

[11] Garry E, Ouattara G, Williams P, Pesta M. 2009. Enumerating chromogenic agar plates using the Color QCount automated colony counter. J Rapid Methods Autom Microbiol 17:46–54. doi:10.1111/j.1745-4581.2008.00150.x.

[12] Putman M, Burton R, Nahm MH. 2005. Simplified method to automatically count bacterial colony forming unit. J Immunol Methods 302:99–102. doi:10.1016/j.jim.2005.05.003. 16002082

[13] Smith TAD, Cabello G, Mingarelli M. 2019. Use of an imaging station for rapid colony counting in radiobiology studies. Appl Radiat Isot 152:106–108. doi:10.1016/j.apradiso.2019.06.028. 31280103

[14] Kashyap R, Roucourt B, Lembo F, Fares J, Carcavilla AM, Restouin A, Zimmermann P, Ghossoub R. 2015. Syntenin controls migration, growth, proliferation, and cell cycle progression in cancer cells. Front Pharmacol 6:241. doi:10.3389/fphar.2015.00241. 26539120 PMC4612656

[15] Brugger SD, Baumberger C, Jost M, Jenni W, Brugger U, Muhlemann K. 2012. Automated counting of bacterial colony forming units on agar plates. PLoS One 7:e33695. doi:10.1371/journal.pone.0033695. 22448267 PMC3308999

[16] Mansberg HP. 1957. Automatic particle and bacterial colony counter. Science 126:823–827. doi:10.1126/science.126.3278.823. 13467282

[17] Goss WA, Michaud RN, McGrath MB. 1974. Evaluation of an automated colony counter. Appl Microbiol 27:264–267. doi:10.1128/am.27.1.264-267.1974. 4589134 PMC380003

[18] Clarke ML, Burton RL, Hill AN, Litorja M, Nahm MH, Hwang J. 2010. Low-cost, high-throughput, automated counting of bacterial colonies. Cytometry A 77:790–797. doi:10.1002/cyto.a.20864. 20140968 PMC2909336

[19] Marotz J, Lubbert C, Eisenbeiss W. 2001. Effective object recognition for automated counting of colonies in Petri dishes (automated colony counting). Comput Methods Programs Biomed 66:183–198. doi:10.1016/s0169-2607(00)00128-0. 11551392

[20] Chiang PJ, Tseng MJ, He ZS, Li CH. 2015. Automated counting of bacterial colonies by image analysis. J Microbiol Methods 108:74–82. doi:10.1016/j.mimet.2014.11.009. 25451456

[21] Wang J, Langer S. 1997. A brief review of human perception factors in digital displays for picture archiving and communications systems. J Digit Imaging 10:158–168. doi:10.1007/BF03168838. PMC3452987 9399169

[22] Brodsky MH, Ciebin BW, Schiemann DA. 1979. A critical evaluation of automatic bacterial colony counters. J Food Prot 42:138–143. doi:10.4315/0362-028X-42.2.138. 30812341

[23] Kramer JM, Kendall M, Gilbert RJ. 1979. Evaluation of the spiral plate and laser colony counting techniques for the enumeration of bacteria in foods. Eur J Appl Microbiol Biotechnol 6:289–299. doi:10.1007/BF00508101.

[24] Manninen MT, Fung DYC, Hart RA. 1990. Spiral system and laser colony scanner for enumeration of microorganisms. J Food Saf 11:177–187. doi:10.1111/j.1745-4565.1990.tb00049.x.

